# Hsa_circ_0001859 Regulates ATF2 Expression by Functioning as an MiR-204/211 Sponge in Human Rheumatoid Arthritis

**DOI:** 10.1155/2018/9412387

**Published:** 2018-01-17

**Authors:** Bingrui Li, Nianyu Li, Le Zhang, Kai Li, Yingtian Xie, Meilan Xue, Zheng Zheng

**Affiliations:** ^1^Medical College of Qingdao University, Qingdao 266071, China; ^2^BGI-Shenzhen, Shenzhen 518083, China; ^3^College of Science, Northeastern University, Boston, MA 02115, USA

## Abstract

**Background:**

circRNAs are part of the competitive endogenous RNA network, which putatively function as miRNA sponges and play a crucial role in the development of numerous diseases. However, studies of circRNAs in rheumatoid arthritis (RA) disease are limited. This work aims to identify the expression pattern of circRNAs in synovial tissues and their inflammatory regulation mechanism.

**Methods:**

We first compared the mRNA expression in rheumatoid arthritis patients with that in healthy volunteers by GEO database mining to identify gene loci specifically expressed in synovial tissues. Functional enrichment algorithms were then used to draw the interactome diagram of circRNAs-miRNAs-mRNAs. Finally, loss-of-function and rescue assays of the candidate circRNAs were performed *in vitro*.

**Results:**

A total of 29 differentially expressed circRNAs related to rheumatoid arthritis were discovered. Silencing of hsa_circ_0001859 suppressed ATF2 expression and decreased inflammatory activity in SW982 cells. Hsa_circ_0001859 could compete with ATF2 for miR-204/211.

**Discussion:**

These findings indicate that hsa_circ_0001859 participates deeply in the process of chronic inflammatory disease in synovial tissue.

## 1. Introduction

As a chronic inflammatory disease, rheumatoid arthritis (RA) involves both genetic and environmental factors. It is characterized by a chronic inflammatory response, cartilage resorption, bone destruction, and bone fibrosis [1]. Circular RNAs (circRNAs) are a category of endogenous RNAs mainly composing transcripts from exons formed by noncollinear reverse splicing. circRNAs are widely expressed in human cells and involved in posttranscriptional regulation of gene expression [2]. Several circRNAs regulate gene expression by acting as microRNA (miRNA) sponges; the circRNAs sequester miRNAs to terminate regulation of their target genes [3]. In 2016, Dou and his colleagues quantified the expression of circRNAs and annotated their potential differential functions during different stages of osteoclastogenesis [4]. Early this year, Ouyang's team proved that several circRNAs that are potential biomarkers for RA patient diagnosis are increased in peripheral blood mononuclear cells from RA patients [5]. However, few studies have identified circRNAs in synovial tissues and elucidated the mechanism of circRNAs in inflammatory regulation.

In our study, we provide evidence that hsa_circ_0001859 promotes the level of inflammation in SW982 cells. Mechanically, hsa_circ_0001859 directly inhibits the miR-204/211 family like a molecular sponge. Silencing of hsa_circ_0001859 by small interfering RNAs (siRNAs) increased miR-204/211 expression and subsequently downregulated the miRNA target, ATF2, to suppress its function and thus promote inflammation in SW982 cells.

## 2. Materials and Methods

### 2.1. Data Mining and Computational Analysis

To explore expression differences between normal and RA cells, we first screened datasets that met the needs of our design by literature mining in the Gene Expression Omnibus (GEO) database. “Rheumatoid” and “arthritis” were searched as keywords in the GEO database, and the list of GSEs is shown in Supplementary Table S1. GSE2053 is the only dataset generated from human RA sample assays. Datasets from other species or other tissues were filtered out. The GEOquery and limma R packages were then used to screen the differentially expressed genes in RA (RA-DEGs). Functional enrichment analyses conducted by DAVID and GeneCards [6, 7], which are based on gene ontology (GO) terms and pathway databases, were applied to cluster the top 500 scored RA-DEGs. miRWalk was used to predict the miRNAs that may downregulate the mRNAs of RA-DEGs (RA-miRNAs), and the interactions between candidate circRNAs and miRNAs were predicted by the StarBase v2.0 tool. Molecular interaction networks were constructed according to mRNA profiling analysis, miRNA target prediction, and functional clustering. Algorithms (TargetScan, miRanda, Pictar2, PITA, and RNA22) provided by open-source databases were used to examine the significance of the correlations of expression between interacting molecules. The degree of correlation determined the gene's functional importance in this circRNA-miRNA-mRNA (CMM) network diagram.

### 2.2. Luciferase Reporter Assays for MicroRNA Targeting

The SW982 cell line (ATCC, USA) was maintained in DMEM cell culture medium (Invitrogen, USA) supplemented with 10% fetal bovine serum (HyClone, USA), 100 units/ml penicillin, and 100 units/ml streptomycin. A synthetic 3′-UTR of ATF2 with the putative miR-204/211 seed region was inserted into the multiple cloning site of the pmiR-RB-Report (Thermo Fisher, USA) vector. SW982 cells were cotransfected individually with wild-type 3′-UTR or the mutant sequence and miR-204 mimics (RiboBio, Guangzhou, China) or a small RNA negative control. We collected the lysates 36 hours after transfection and evaluated the activity of renilla luciferase according to the manual.

### 2.3. RNA Interference and Transfection

siRNAs targeting candidate circRNAs (siRNA-circ0001859), the siRNA-negative control, and the siRNA-positive control were synthesized commercially (RiboBio, Guangzhou, China). The functional sequence of siRNA-circ0001859 is 5′-TGTTTCCCT-3′. The SW982 cell line was transfected with siRNA-circ0001859 using Lipofectamine 2000 (Invitrogen, USA) and cultured in DMEM in a 96-well plate. Before transfection, the medium was replaced with antibiotic-free medium and cultured for a whole day. Then, the RNA-interferon-Lipofectamine 2000 complex was added to each well and incubated for 48 hours at 37°C with normal DMEM.

### 2.4. Luciferase Reporter Assays for circRNA Rescue

MiR-204 pEGFP-C1 vector encoding the enhanced green fluorescent protein gene (GeneCopoeia, Guangzhou, China) was transfected into SW982 cells. Empty vector, circ_0001859, siRNA-circ0001859, miR-204/211 inhibitor, or miRNA inhibitor control was then cotransfected into the SW982 cells. All small RNA reagents were commercially available (RiboBio, Guangzhou, China). Cell harvesting and luciferase assays were performed as described above.

### 2.5. Real-Time Quantitative PCR

Total RNA was isolated from SW982 cells using TRIzol reagent (Thermo Fisher, USA). For mRNA analysis, total RNA was reverse transcribed using random primers. For miRNA, quantitative PCR was performed by using the Bulge-Loop miRNA primer set (RiboBio, Guangzhou, China) for specific miRNA transcription. mRNA expression levels were measured relative to GAPDH, whereas miRNA expression levels are reported relative to U6. The primers used in this work are presented in Supplementary Table S2.

### 2.6. Western Blotting

Lysis buffer (50 mM Tris-HCl, pH 7.4, 150 mM NaCl, 1% Nonidet P40, and 0.1% sodium dodecyl sulfate) was used for protein purification. The concentration was determined using a bicinchoninic acid protein kit (Thermo Fisher, USA). Anti-TNF antibody (1 : 500 dilution), anti-ATF2 antibody (1 : 2000 dilution), anti-IL-6 antibody (1 : 4000 dilution), anti-IL-1*β* antibody (1 : 3000 dilution), anti-c-Fos antibody (1 : 4000 dilution), and anti-c-Jun antibody (1 : 3000 dilution) were used according to standard protocols. All antibodies were purchased from Abcam, UK. GAPDH was applied as an internal control.

### 2.7. Statistical Analysis

Statistically significant differences between multiple groups were calculated by one-way ANOVA. Bartlett's test for equal variances and Dunnett's test for multiple comparisons were both applied as subsequent statistical methods. Results are reported as the mean ± SEM. *P* values of less than 0.05 were considered statistically significant. GraphPad Prism 6.0 software was used to perform data analysis and visual display.

## 3. Results

### 3.1. The Expression Profile in Rheumatoid Arthritis Is Unique

**Figure 1:**
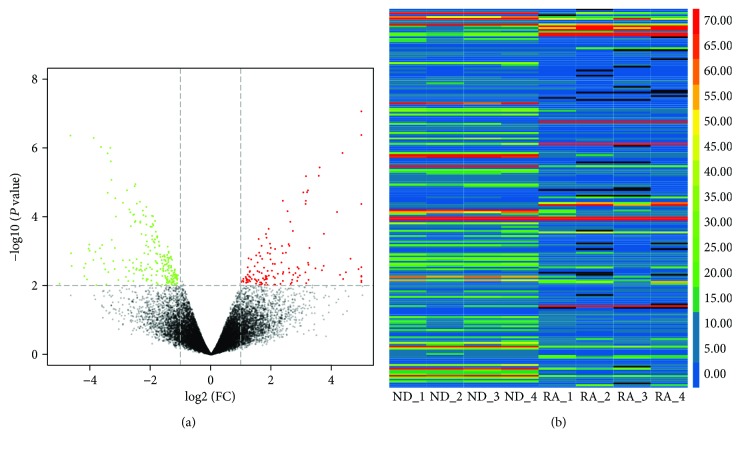
Differential expression of genes in RA synovial tissues. (a) The fold value and *P* value were used to build the volcano plots. The horizontal line corresponds to twice the normal and downward adjustment between the normal and RA samples, and the vertical line shows the *P* value. The red points and green points in the plot represent the total of 369 DEGs with statistical significance. (b) Hierarchical clustering analysis of DEGs. Each group has four components (greater than two-fold expression difference; *P* < 0.05). The expression values are expressed in different colors to indicate expression above or below the median expression level for all samples.

The variation of gene expression between the normal and RA cartilage samples was shown in volcano plots (Figure 1(a)). Hierarchical clustering illustrated the gene expression profile patterns in normal and RA cartilage samples (Figure 1(b)). The top 500 differentially expressed genes (DEGs) are summarized in Supplementary Table S3. Using a combination of methods for significant differential expression analysis, we finally identified 141 upregulated and 228 downregulated genes in RA versus normal synovial tissues.

To examine the biological significance, the list of identified DEGs was uploaded into DAVID and GeneCards for GO term annotation and pathway analysis. The signaling pathways highly enriched to DEGs were recognized and mapped by the DAVID and GeneCards annotation clustering services. Rheumatoid arthritis was identified as the highest scored related disease based on functional analysis, and the enriched pathways were the IL-2 signaling pathway, SMAD signaling network, and MAPK signaling pathway (Supplementary Table S4). SMAD3, ATF2, and JUNB were among the top directly related genes in these RA relative pathways (Supplementary Table S5).

### 3.2. ATF2 Is Upregulated and Correlates with Inflammatory Hyperfunction Characteristics of RA Tissue

**Figure 2:**
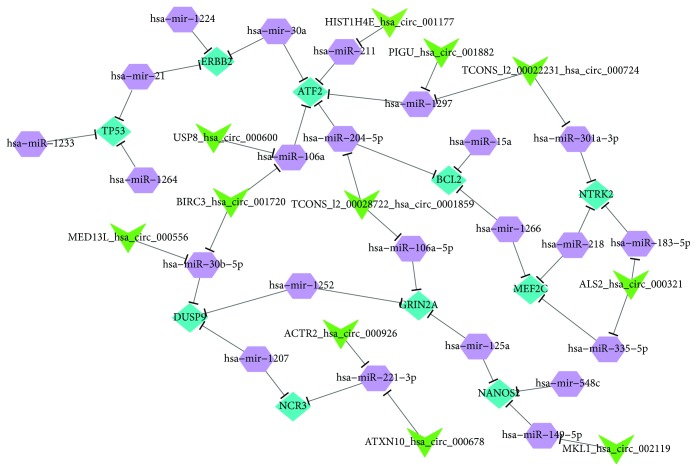
circRNA-miRNA-mRNA network. The network illustrates 44 molecules, in which diamonds represent mRNA, hexagons represent miRNA, and triangles indicate circRNA. The interactions between them are shown by edges.

In this study, we performed functional clustering and pathway analysis of the top 500 DEGs immediately after the expression profile assay of GSE2053. Then, miRNA-mRNA (DEG) interactions were predicted and evaluated using miRWalk 2.0. The interactions between miRNAs and circRNAs were predicted by the StarBase v2.0 tool. Based on the results from these two prediction tools, the functional interaction graph (CMM network) (Figure 2) was output using the spring embedder algorithm in Cytoscape [8]. The spring embedder algorithm is based on the establishment of a mechanical system in which the edges of the graph correspond to the springs. The graph can show the interaction of the high scores of circRNAs, miRNAs, and mRNAs in the functional analysis. The low scores in the functional clustering are filtered out. For our CMM networks, most of the nodes were connected to each other, although some modified genes were isolated because they had no partners. The “central” gene is a gene with multiple margins, which indicates that the gene interacts positively with many of the partner molecules. In conjunction with the CMM network and the results in Supplementary Table S5, ATF2 was identified as the only coincident gene. Important roles of ATF2 in pathways associated with RA, such as the Toll-like pathway and p38 MAPK, were detected.

Four miRNAs (miR-204-5p, miR-30a, miR-106a, and miR-211) were identified as linked to the ATF2 gene in the CMM networks. However, miR-204/211 was more statistically significant than the other three miRNAs. In additional, literature evidence showed that miR-204/211 might have a crucial function in bone remodeling and could inhibit the STAT3 protein, which has been identified as an important molecule for the survival of RA synovial fibroblasts [9, 10].

### 3.3. ATF2 Is a Functional Target of miR-204/211 in SW982 Cells

**Figure 3:**
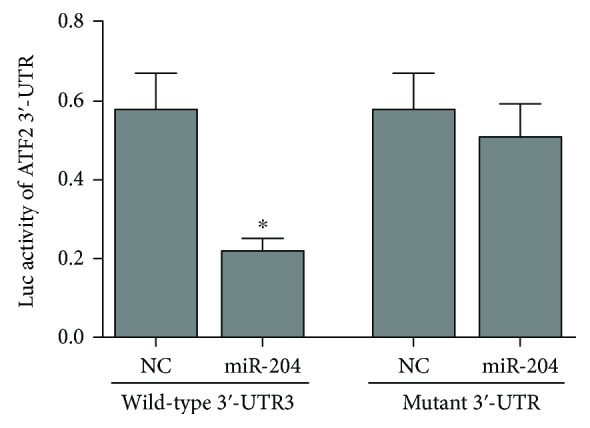
MiR-204/211 targeting of ATF2 mRNA. Luciferase reporter analysis of wild-type or mutant ATF2 3′-UTR. MiR-204 was transfected with wild-type or mutant vectors. The values are the mean ± SEM of three replicates. ^∗^ *P* < 0.05.

Based on the previous computational studies, ATF2 was selected as the most RA-relevant gene in this interaction network, and the miR-204/211 family was selected as a potential miRNA regulator in RA progression at the epigenetic level. Thus, we cloned the 3′-UTR fragments of ATF2 containing hsa-miR-204-5p (miR-204) binding sites and their mutant fragments into the pmiR-RB-Report vector to perform functional assays. Here, miR-204 represents the mature sequence of the miR-204/211 family. Based on the endogenous expression of miR-204/211 in the SW982 cell line, we transfected different constructs into SW982 cells (Figure 3). The results showed that only the cells transfected with the 3′-UTR of ATF2 and miR-204 exhibited significantly lower luciferase activity. These results demonstrate that the miR-204/211 family can directly target the ATF2 gene in SW982 cells by interacting with the 3′-UTR region.

### 3.4. Hsa_circ_0001859 Inhibits the Transcription Activity of miR-204/211

**Figure 4:**
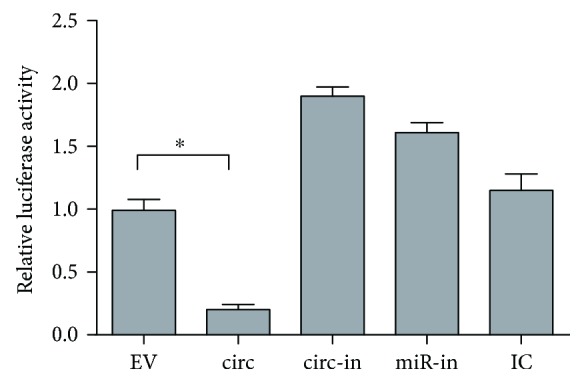
Circ_0001859 inhibits the transcription activity of miR-204. Effects of empty vector (EV), circ_0001859, circ_0001859 inhibitor (circ-in), miR-204/211 inhibitor (miR-in), and miRNA inhibitor control (IC) on the luciferase activity of miR-204 in SW982. ^∗^ *P* < 0.05.

To screen circRNAs that may be regulated by miR-204/211, we profiled the StarBase v2.0 database and identified 29 circRNAs with binding sites for miR-204/211 (Supplementary Table S6). Visualization analysis based on the spring embedder algorithm further verified these circRNAs (Figure 2). In addition, only hsa_circ_0001859 was identified by both methods, and it received the highest score. It also was at the critical position of the CMM visualization map and was closely related to multiple pathogenic pathways of RA according to the previous functional analysis. Indeed, reduction of luciferase activity was observed when hsa_circ_0001859 and miR-204 were cotransfected into cells simultaneously (Figure 4). Thus, we speculated that hsa_circ_0001859 can target miR-204/211 to inhibit its expression and hsa_circ_0001859 affects ATF2 expression by acting as a competing endogenous RNA (ceRNA) in the chronic inflammatory response.

### 3.5. Hsa_circ_0001859 Promotes ATF2 Expression by Acting as a ceRNA

**Figure 5:**
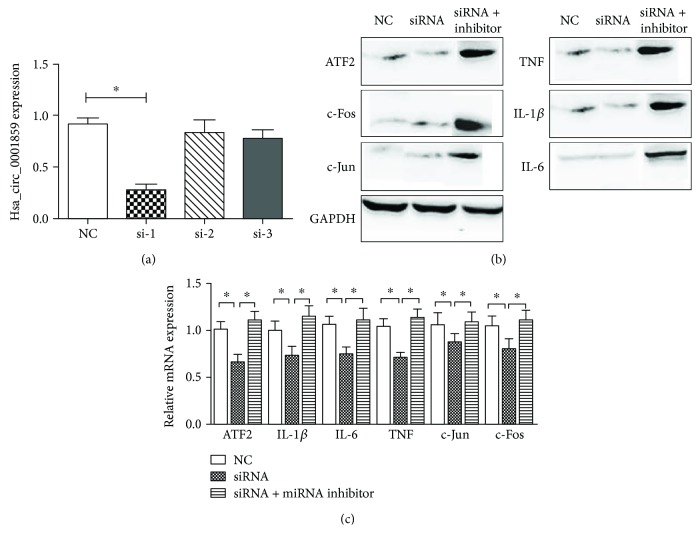
Effects of hsa_circ_0001859 on gene expression in SW982 cells. (a) The influence of three different siRNAs against hsa_circ_0001859 was analyzed by quantitative polymerase chain reaction (qPCR), and siRNA-circ0001859-1 (si-1) exhibited the best inhibitory effect. (b) The expression levels of ATF2, c-Jun, c-Fos, TNF, IL-1*β*, and IL-6 were analyzed by Western blotting. The values are the mean ± SEM of three replicates. *P* < 0.05. GAPDH was used as a loading control. (c) ATF2, c-Jun, c-Fos, TNF, IL-1*β*, and IL-6 mRNA expression levels were analyzed after knockdown of hsa_circ_0001859 by siRNA-circ0001859 or cotransfection with siRNA-circ0001859 and the miR-204/211 inhibitor. ^∗^ *P* < 0.05.

To analyze the influence of hsa_circ_0001859 on inflammatory activity, SW982 cells were transfected with siRNAs specific for hsa_circ_0001859 (si-1) to examine the effect of suppression of hsa_circ_0001859. In response to this inhibition, ATF2 mRNA expression decreased (Figure 5(a)), and the expression of c-Jun and c-Fos, which are activated by ATF2 in inflammation as downstream signals, also decreased (Figures 5(b) and 5(c)). We hypothesized that hsa_circ_0001859 acts as a ceRNA to induce inflammatory activity in synovial tissue and regulate ATF2 expression. To confirm that the miR-204/211 inhibitor could reverse ATF2 repression via hsa_circ_0001859 knockdown, siRNA-circ0001859 and miR-204/211 inhibitor were cotransfected into SW982 cells. The expression of ATF2, c-Jun, and c-Fos was rescued, and the elimination of the effects of hsa_circ_0001859 was further confirmed by the low expression of IL-1*β*, IL-6, and TNF (Figures 5(b) and 5(c)).

### 3.6. Hsa_circ_0001859 Regulates Inflammation in SW982 Cells via ATF2

**Figure 6:**
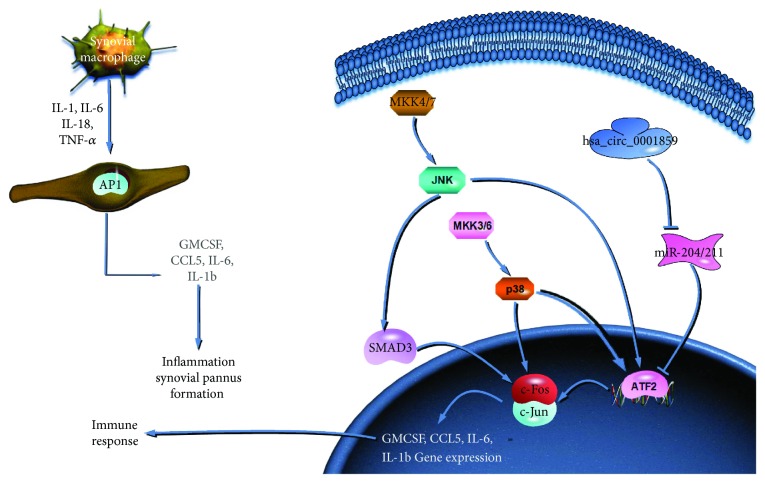
RA-related molecular signaling pathway. ATF2 is involved in the TLR signaling and TNF pathways. ATF2 regulates transcriptions with the c-Fos protein to regulate the expression of GMCSF, CCL5, IL-6, and IL-1b. Hsa_circ_0001859 can also regulate ATF2 by inhibiting miR-204/211.

We performed quantitative RT-PCR and Western blotting to assess the expression of ATF2, IL-1*β*, IL-6, TNF, c-Fos, and c-Jun at the protein level. ATF2 expression was clearly decreased by siRNA-circ0001859 treatment, and this decrease was reversed by cotransfection with siRNA-circ0001859 and miR-204/211 inhibitor in SW982 cells (Figure 5(c)). Additionally, the mRNA and protein expression of IL-1*β*, IL-6, and TNF was significantly decreased by siRNA-circ0001859 treatment, and these effects were reduced by cotransfection with siRNA-circ0001859 and miR-204/211 inhibitor in SW982 cells (Figure 5(b)). IL-6, TNF, and IL-1*β* are downstream signaling molecules in the RA pathway and have been related to inflammation activity (Figure 6).

## 4. Discussion

Both exonic and intronic circRNAs are widely expressed in mammalian cells and show potential function in gene regulation [11]. Their expression levels can be more than 10 times higher than that of their linear isomers [12]. Their high conservation and stability in cells may lead circRNAs to be used as ideal biomarkers and potential therapeutic targets compared to other noncoding RNAs, such as miRNAs and lncRNAs [13]. Considerable evidence indicates that these sequences play roles as miRNA “sponges” in vivo [3].

Recent studies have made significant progress in the apparent genetic regulation of RA pathogenesis and potential therapeutic targets such as miRNAs and lncRNAs [14]. However, the occurrence of circRNA in synovial tissues has been largely uncharacterized. This study is the first functional assay based on computational enrichment to characterize circRNA expression patterns in human synovial tissue. In this study, we identified many abnormally expressed circRNAs in RA compared with normal synovial tissue. Functional loss and rescue experiments confirmed that hsa_circ_0001859 plays a key role in aggravating the inflammatory process of SW982 cells.

We assumed that hsa_circ_0001859 functions as a decoy for ATF2 through the “microRNA-sponge” mechanism. Our results showed that miR-204/211 binds the 3′-UTR of ATF2 and that hsa_circ_0001859 can shelter miR-204/211 binding sites. The seed region sequence of this circRNA-miRNA-mRNA interaction is 5′-UUCCCUUU-3′.

Several studies have reported that miR-204/211 may have a crucial function in bone remodeling by inhibiting Wnt signaling pathways. It can also promote fat formation in human adipose-derived mesenchymal stem cells and affect synovial inflammation development [9]. miR-204 has also been reported to inhibit the STAT3 protein, which is an important molecule for the survival of RA synovial fibroblasts, and this effect can be reversed by the corresponding anti-miRNA [10].

These findings suggest that the mechanism of circRNAs is complex and that other molecules are involved in synovial degradation. In our study, we only focused on miR-204/211 as a candidate miRNA cluster that may target hsa_circ_0001859 and ATF2. ATF2 is a member of the CREB family, which is usually associated with AP1 (c-Jun and c-Fos) and heterodimers. Activation of ATF2 usually requires phosphorylation of other kinases, such as JNK, ERK, or p38 MAPK [15, 16]. ATF2 plays an important role in the skeletal system, central nervous system, and inflammation and is involved in oncogenic transformation, adaptive responses of the cell to viral infections, and toxic stresses [17, 18].

In this study, we first performed a bioinformatic prediction of the circRNA-miRNA-mRNA network, which indicated the potential associations between the circRNA and its target gene. The network also provides a crucial reference for discovering the interactions of other candidate genes and their potential targets. We also confirmed that hsa_circ_0001859 regulates ATF2 expression and is involved in the SW982 cell inflammatory process.

Based on the results from several other studies, more than one signaling pathway is involved. One of these superfamilies is the MAPK pathway. This pathway is important for inflammation and cell apoptosis and has been implicated in the pathogenesis of RA [19]. In this pathway, MKK4/7 specifically activates JNK. After JNK is activated, it enhances the transcription activity of Fos/Jun (AP1) by phosphorylating ATF2. In addition, when p38 is phosphorylated by MKK3/6, it can activate AP1 directly in the pathway [20]. AP1 is also activated in the TLR pathway, and this pathway activates TNF-*α*, IL-1*β*, IL-6, and IL-12. There is a significant correlation between AP1 and ATF2 expression in RA synovial tissue [21, 22]. High levels of activated IL-2 and TNF-*α* are observed in RA synovial tissue [23]. In summary, the MAPK and TLR pathways have different functions but both may target ATF2 for regulation in human cells, and thus, hsa_circ_0001859 may influence these pathways by regulating ATF2.

In summary, the comparison of the expression profiles revealed that 29 circRNAs were differentially expressed between RA and normal tissues. The functional analysis showed that circRNA is an important factor in this difference in gene expression. We suggest that hsa_circ_0001859 functions as a decoy to regulate ATF2 expression by acting as a sponge to directly inhibit miR-204/211 transcription. Silencing of hsa_circ_0001859 by siRNA suppressed ATF2 expression and reduced the inflammation level in SW982 cells. Our study supports the application of hsa_circ_0001859 as a potential-specific siRNA target in RA therapy and a new approach for treating RA by targeting genes involved in pathogenesis.

## Additional Points

*Statement*. Gene expression data were acquired from the National Center for Biotechnology Information (NCBI) Gene Expression Omnibus (GEO) database (accession number GSE2053); we will protect the privacy rights of patients' information. SW982 cells were purchased from the American type culture collection.

## Supplementary Materials

Supplementary 1 Table S1: the list of all GSEs.

Supplementary 2 Table S2: the primers used in this work.

Supplementary 3 Table S3: the top 500 differentially expressed genes (DEGs).

Supplementary 4 Table S4: GeneAnalytics disease and pathway.

Supplementary 5 Table S5: the top directly related genes in RA relative pathways.

Supplementary 6 Table S6: circRNAs with binding sites for miR-204/211.

## References

[1] McInnes I. B., Schett G. (2007). Cytokines in the pathogenesis of rheumatoid arthritis. *Nature Reviews Immunology*.

[2] Memczak S., Jens M., Elefsinioti A. (2013). Circular RNAs are a large class of animal RNAs with regulatory potency. *Nature*.

[3] Hansen T. B., Jensen T. I., Clausen B. H. (2013). Natural RNA circles function as efficient microRNA sponges. *Nature*.

[4] Dou C., Cao Z., Yang B. (2016). Changing expression profiles of lncRNAs, mRNAs, circRNAs and miRNAs during osteoclastogenesis. *Scientific Reports*.

[5] Ouyang Q., Wu J., Jiang Z. (2017). Microarray expression profile of circular RNAs in peripheral blood mononuclear cells from rheumatoid arthritis patients. *Cellular Physiology and Biochemistry*.

[6] Huang D. W., Sherman B. T., Lempicki R. A. (2009). Systematic and integrative analysis of large gene lists using DAVID bioinformatics resources. *Nature Protocols*.

[7] Stelzer G., Rosen N., Plaschkes I. (2016). The GeneCards suite: from gene data mining to disease genome sequence analyses. *Current Protocols in Bioinformatics*.

[8] Shannon P., Markiel A., Ozier O. (2003). Cytoscape: a software environment for integrated models of biomolecular interaction networks. *Genome Research*.

[9] Collino F., Bruno S., Deregibus M. C., Tetta C., Camussi G. (2011). MicroRNAs and mesenchymal stem cells. *Vitamins & Hormones*.

[10] Xie Y.-f., Shu R., Jiang S.-y., Liu D.-l., Zhang X.-l. (2011). Comparison of microRNA profiles of human periodontal diseased and healthy gingival tissues. *International Journal of Oral Science*.

[11] Jeck W. R., Sharpless N. E. (2014). Detecting and characterizing circular RNAs. *Nature Biotechnology*.

[12] Li P., Chen S., Chen H. (2015). Using circular RNA as a novel type of biomarker in the screening of gastric cancer. *Clinica Chimica Acta*.

[13] Ling H., Fabbri M., Calin G. A. (2013). MicroRNAs and other non-coding RNAs as targets for anticancer drug development. *Nature Reviews Drug Discovery*.

[14] Esteller M. (2011). Non-coding RNAs in human disease. *Nature Reviews Genetics*.

[15] Xia Z., Dickens M., Raingeaud J., Davis R. J., Greenberg M. E. (1995). Opposing effects of ERK and JNK-p38 MAP kinases on apoptosis. *Science*.

[16] Karin M. (1995). The regulation of AP-1 activity by mitogen-activated protein kinases. *Journal of Biological Chemistry*.

[17] Lopez-Bergami P., Lau E., Ronai Z.’e. (2010). Emerging roles of ATF2 and the dynamic AP1 network in cancer. *Nature Reviews Cancer*.

[18] Gupta S., Campbell D., Derijard B., Davis R. J. (1995). Transcription factor ATF2 regulation by the JNK signal transduction pathway. *Science*.

[19] Schett G., Zwerina J., Firestein G. (2008). The p38 mitogen-activated protein kinase (MAPK) pathway in rheumatoid arthritis. *Annals of the Rheumatic Diseases*.

[20] Raingeaud J., Whitmarsh A. J., Barrett T., Derijard B., Davis R. J. (1996). MKK3- and MKK6-regulated gene expression is mediated by the p38 mitogen-activated protein kinase signal transduction pathway. *Molecular and Cellular Biology*.

[21] Kawai T., Akira S. (2007). Signaling to NF-*κ*B by Toll-like receptors. *Trends in Molecular Medicine*.

[22] Han Z., Boyle D. L., Chang L. (2001). c-Jun N-terminal kinase is required for metalloproteinase expression and joint destruction in inflammatory arthritis. *The Journal of Clinical Investigation*.

[23] Feldmann M., Brennan F. M., Maini R. N. (1996). Role of cytokines in rheumatoid arthritis. *Annual Review of Immunology*.

